# Rapid overview of systematic reviews of nocebo effects reported by patients taking placebos in clinical trials

**DOI:** 10.1186/s13063-018-3042-4

**Published:** 2018-12-11

**Authors:** Jeremy Howick, Rebecca Webster, Nigel Kirby, Kerry Hood

**Affiliations:** 1 Nuffield Department of Primary Care Health Sciences, University of Oxford, Radcliffe Primary Care Building, Oxford, OX2 6GG UK; 2 Department of Psychological Medicine, King’s College London, 3rd Floor Weston Education Centre, 10 Cutcombe Road, Denmark Hill, London, SE5 9RJ UK; 3 Centre for Trials Research, College of Biomedical & Life Sciences, Cardiff University, 7th Floor, Neuadd Meirionnydd, Heath Park, Cardiff, CF14 4YS UK

**Keywords:** Placebo, Nocebo, Adverse events, Systematic review, Randomized trial

## Abstract

**Background:**

Trial participants in placebo groups report experiencing adverse events (AEs). Existing systematic reviews have not been synthesized, leaving questions about why these events occur as well as their prevalence across different conditions unanswered.

**Objectives:**

1. To synthesize the evidence of prevalence of AEs in trial placebo groups across different conditions.
2. To compare AEs in trial placebo groups with AEs reported in untreated groups within a subset of randomized trials.

**Search methods:**

We searched PubMed for records with the word “nocebo” in the title and “systematic” in any field. We also contacted experts and hand-searched references of included studies.

**Study eligibility:**

We included any systematic review of randomized trials where nocebo effects were reported. We excluded systematic reviews of non-randomized studies.

**Participants and interventions:**

We included studies in any disease area.

**Study appraisal and synthesis methods:**

We appraised the quality of the studies using a shortened version of the Assessment of Multiple Systematic Reviews tool (AMSTAR) tool. We reported medians and interquartile ranges (IQRs) of AEs. Among the trials within the review that included untreated groups, we compared the prevalence of AEs in untreated groups with the prevalence of AEs in placebo groups.

**Results:**

We identified 20 systematic reviews. These included 1271 randomized trials and 250,726 placebo-treated patients. The median prevalence of AEs in trial placebo groups was 49.1% (IQR 25.7–64.4%). The median rate of dropouts due to AEs was 5% (IQR 2.28–8.4%). Within the 15 of trials that reported AEs in untreated groups, we found that the AE rate in placebo groups (6.51%) was higher than that reported in untreated groups (4.25%).

**Limitations:**

This study was limited by the quality of included reviews and the small number of trials that included untreated groups.

**Conclusions and implications of key findings:**

AEs in trial placebo groups are common and cannot be attributed entirely to natural history. Trial methodologies that reduce AEs in placebo groups while satisfying the requirement of informed consent should be developed and implemented.

**Electronic supplementary material:**

The online version of this article (10.1186/s13063-018-3042-4) contains supplementary material, which is available to authorized users.

## Background

Some recent systematic reviews suggest that trial participants who are allocated to placebo groups experience adverse events (AE), including AEs attributed to apparent drug interactions [1–6]. Yet adverse drug reactions cannot be directly caused by placebo treatments. There are two overlapping explanations for how this might occur. First, a patient may have an underlying condition whose natural history produces some event (such as a headache), then the patient *misattributes* the event to the trial intervention (in their case, a placebo). Second, having been warned about side effects in the patient information sheets (or elsewhere), the patient may expect an AE. This negative expectation could then produce the event [7]. There is some empirical support for the latter explanation. In one multicenter randomized trial of aspirin or sulfinpyrazone for treating unstable angina, due to differences in individual hospital review processes, patients either received or did not receive a statement outlining possible gastrointestinal side effects. This resulted in a sixfold increase (*P* < 0.001) in the number of individuals withdrawing from the study because of subjective, minor gastrointestinal symptoms [8]. Major (“objective”) complications such as peptic ulcer or bleeding as diagnosed by study physicians were similar across centers.

There are two gaps in the literature about nocebo effects within clinical trials. First, the systematic reviews in the area have not been synthesized. This leaves questions about the comparative prevalence of AEs within placebo groups across different conditions unanswered. Second, the existing systematic reviews often conflate natural history with nocebo effects. The fact that an AE occurs after taking a placebo does not imply that the AE was caused by the placebo. This mistaken inference was noted in studies investigating positive placebo effects > 20 years ago [9]. It was resolved by comparing what happens within three-armed trials (treatment, placebo, no treatment) to patients who receive a placebo treatment with patients who are left untreated. The same method could also be used to differentiate between nocebo effects and natural history.

## Objectives

In this rapid systematic review, we addressed both of these gaps and:

1. synthesized the systematic reviews of nocebo effects; and
2. reviewed trials within these systematic reviews that reported AEs in untreated control groups to compare these with AEs in placebo groups.

## Methods

### Protocol and registration

The protocol for this rapid review was published on PROSPERO on 05/04/18 (record no. CRD42018092437).

### Eligibility criteria

We included any systematic review of randomized trials where AEs were reported and quantified. We included systematic reviews of trials in any condition. We excluded reviews of non-randomized trials, and reviews that did not include quantitative data about AEs within trial control groups.

### Information sources

We searched for PubMed records with the word “nocebo” in the title and “systematic” in any field (see Appendix). We also contacted experts and searched references of included studies.

### Study selection and data collection

Two authors (JH, NK) extracted data from the systematic reviews: about year of publication, authors, disease area, type of AE, and rates of AEs.

Two authors (NK, RW) extracted data from the trials within the systematic reviews to identify those which included AE rates within untreated groups and compared these with AEs (in the same trial) in placebo groups. They reported AE rates in all three groups where these were found. One author (JH) acted as a second extractor for a random selection (*n* = 50) of the studies. Discrepancies were resolved by discussion between review authors.

### Risk of bias in individual studies

To ensure a minimum level of quality within included reviews, we assessed whether the following two items of the Assessment of Multiple Systematic Reviews tool (AMSTAR) were satisfied: *were two or more electronic sources searched?*; and *was the scientific quality of the included studies assessed and reported?* Other overview authors have used similar assessment criteria [10, 11]. We performed a sensitivity analysis excluding studies which did not meet the two criteria.

### Summary measures

We summarized the median and interquartile ranges (IQR) of AEs, as reported by review authors, as well as drop-out rates. When at least three studies reported specific AEs (such as headache), we reported these, also using medians and IQRs.

In trials within the reviews that reported AE rates in untreated groups, we summarized the rates of AEs in the untreated and placebo groups. To complete this analysis, we needed references to individual trials within the reviews. When such references were not published in the systematic review, we sent the corresponding author an email request. If we did not receive a response, we sent a follow-up email, after which (if we still did not receive a response) we did not search the trials within that review.

### Synthesis of results

We reported the median and IQRs for overall AEs and dropout rates due to AEs. For our analysis of AEs reported in three or more studies, we also reported medians and IQRs. Among the trials within the review that included untreated groups, we compared the prevalence of AEs in untreated groups with the prevalence of AEs in placebo groups.

### Other potential sources of bias

A source of potential bias could have been the method by which the AEs were ascertained. AE evaluation strategies involving structured assessments (checklists of likely AEs) risk confusing misattribution of symptoms caused by the underlying condition to the (placebo) intervention. For example, pain, nausea, and headaches are common symptoms of many diseases. Patients who are asked whether they experienced one of these are therefore likely to say yes because of the underlying condition or simply because these are common. We could not infer from the report whether such an event (when ascertained using structured assessments) was caused by the underlying condition, a negative expectation, or a treatment. Spontaneous strategies for measuring AEs address this problem by not naming potential AEs in checklists and instead asking patients to report any AEs that arise. We compared AEs assessed using the different methods in an exploratory analysis. Data were not suitable for pooling for this analysis so we reported our findings narratively.

## Results

### Study selection

**Fig. 1:**
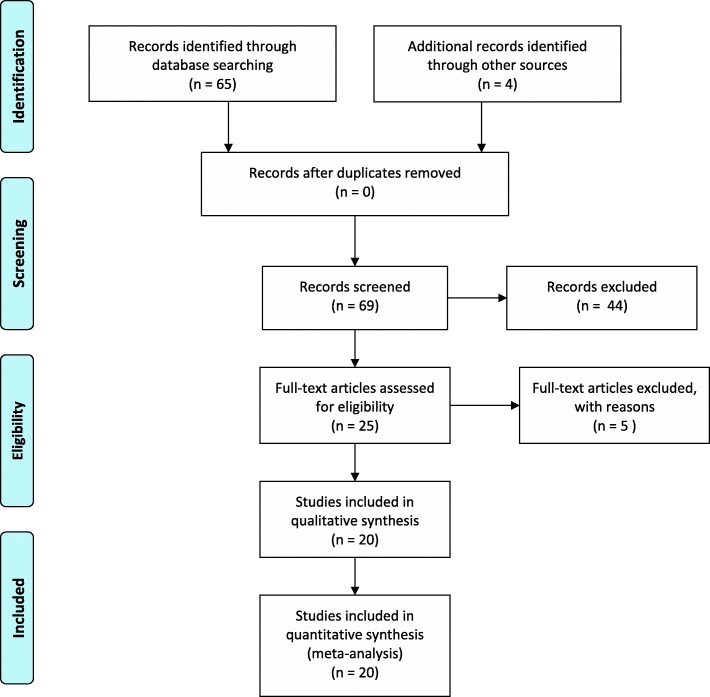
PRISMA diagram records

Our search yielded 20 systematic reviews (see Fig. 1) that met our inclusion criteria [12–31]. A total of 1271 randomized trials and 250,726 placebo-treated patients were included within these reviews.

### Study characteristics

The studies were all published in the last 12 years and pain-related conditions were most commonly studied within the reviews [12–14, 16, 19, 20, 23]. Other conditions were: depression [18, 21, 24, 26]; multiple sclerosis [22]; motor neuron disease [27]; restless leg syndrome [28]; Parkinson’s disease [29]; Alzheimer’s [30]; cardiovascular disease [25]; and epilepsy [31]. Two reviews included trials in any condition [15, 17].

Some of the reviews reported data separately for different types of treatments or conditions without combining the data. Amanzio et al. [12] reported trials of non-steroidal, anti-inflammatory drugs (NSAIDs); triptans, and anticonvulsants separately; Häuser et al. [13] reported data for fibromyalgia and painful diabetic neuropathy trials separately; Mitsikostas et al. [19] reported data for symptomatic and prophylactic trials separately. Papadopoulos et al. [22] reported symptomatic and disease-modifying trials separately, and Rief et al. [25] reported tricyclic (TCA) and selective serotonin reuptake inhibitor (SSRI) trials separately. We followed the authors and did not combine the separately reported data.

Ninety distinct types of AEs were reported. These ranged from abdominal pain and dry mouth to cancer and stroke.

### Risk of bias within studies

Eight reviews [12, 13, 15, 16, 18, 25, 26, 28] met both of our quality criteria (searched at least two databases and reported data about quality of included trials, see Additional file 1: Table S1). Seven reviews met neither quality criteria [19, 20, 22–24, 27, 30]. Of the five studies that met one of our quality criteria, one [14] searched more than one database and four assessed and reported the quality of included studies [17, 21, 29, 31]. There were insufficient data to formally analyze the relationship between review quality and AE data; however, there did not seem to be any correlation between review quality and AEs. Studies which met at least one criteria had the highest rates of AEs (64.7%) compared with studies that met both criteria (36.6)%) and no criteria (54.9%), while the rates of drop out were similar across those which met no (6%), one (4.8%), and both (5.3%) quality criteria.

### Results of individual studies and synthesis of results

The results of individual studies are provided in Table 1. The median prevalence of AEs in trial placebo groups (as defined by the individual trials within the reviews) was 49.1% (IQR 25.7–64.4%). The median rate of dropouts due to AEs was 5% (IQR 2.28–8.4%).

**Table 1:** Summary of adverse effects in placebo groups within trials

Author	Disease	Trials (no patients in placebo group)	Adverse events (%)	Dropouts due to intolerance (%)
Amanzio, 2009	Migraine			
*NSAID placebos*		10 (337)	4.16	1.00
*Triptans placebos*		3 (289)	2.08	0.39
*Anticonvulsant placebos*		6 (142)	5.57	7.71
Häuser, 2012a	Fibromyalgia	58 (5027)	59.90	9.60
	Painful diabetic peripheral neuropathy	62 (5086)	46.20	5.80
Häuser, 2012b	Fibromyalgia syndrome	18 (3546)		10.90
Koog, 2014a	Any treated by acupuncture	58 (2249)	13	1.36
Koog, 2014b	Knee osteoarthritis	281 (22,284)	27	4.80
Mahr, 2017	Any	231 (149,855)	73	5.10
Mahr, 2017 (additional)				
Meister, 2017	Depression	23 (2929)	57.00	4.00
Mitsikostas, 2010	Headaches	56 (n/a)		
*Symptomatic treatments*		n/a (n/a)	18.45	0.33
*Prophylactic treatments*		n/a (n/a)	42.78	4.75
Mitsikostas, 2012	Fibromyalgia	16 (2016)	67.20	9.50
Mitsikostas, 2014	Depression	21 (3255)	44.70	4.50
Papadopoulos, 2010	Multiple sclerosis			
*Symptomatic treatments*		44 (1732)	25.30	2.10
*Disease-modifying treatments*		56 (5623)	74.42	2.34
Papadopoulos, 2012	Neuropathic pain	12 (943)	52	6.00
Rief, 2006	Cardiovascular disease			17.61
Rief, 2009	Depression or anxiety	143 (12,742)		24.70
*TCA studies*				
*SSRI studies*				
Rojas-Mirquez, 2014	Depression	16 (739)	64%	
Shafiq, 2017	Motor neuron disease	12 (1288)	78.30	8.40
Silva, 2017	Restless leg syndrome	72 (5040)	45.36	2.07
Stathis, 2013	Parkinson’s disease	41 (3544)	64.70	8.80
Zis, 2015	Alzheimer’s disease	20 (3049)	57.80	6.60
Zis, 2017	Refractory partial epilepsy (during pre-surgical monitoring)	4 (125)	76.80	3.20
Median (IQR)			49.1% (25.8–64.5%)	5% (2.3–8.4%)

*FMS* fibromyalgia syndrome, *DPN* diabetic peripheral neuropathy

Only some reviews provided details about specific AEs reported within the reviewed trials. In all, 90 different AEs were reported and 17 types of AEs were reported in at least three trials (see Table 2). Eight of these had median rates > 5%: headache (18%); vomiting (7.7%); fatigue (7.11%); insomnia (5.7%); burning (5.88%); somnolescence (5.56%); dizziness (5.1%); and constipation (6.43%).

**Table 2:** Adverse event (AE; %) within placebo groups, by type of AE (where at least three reviews reported the same AE)

	Amanzio, 2009			Mahr, 2017a	Mahr, 2017b	Rief, 2006	Rief, 2009		Rojas-Mirquez, 2014	Median (IQR)
	A^a^	B^b^	C^c^				TCA	SSRI		
Abdominal pain	2.97	1.04	1.04	6.8	3.3		8.4	9	5.68	*4.5 (2.5–7.2)*
Burning	5.88	1.83	8.7							*5.9 (3.9–7.3)*
Chest discomfort/pain	0	0.8		4.2		0.82				*0.8 (0.6–1.7)*
Chills	1.4	0.47	3.7							*1.4 (0.9–2.6)*
Diarrhea		1.25	3.51	7.7		3.53				*3.5 (2.9–4.6)*
Dry mouth	4.26	1.75	3.11	5.1			19.2	6.4		*4.7 (3.4–6.1)*
Dyspepsia	1.13	1.46	3.21	3.9		2.03				*2.0 (1.5–3.2)*
Fatigue	2.85	1.47	8.72	9.3			17.3	5.5		*7.1 (3.5–9.2)*
Insomnia			0	5.7			13.3	11.1	4.73	*5.7 (4.7–11.1)*
Paresthesia	1.1	0.88	6.58	3.6						*2.4 (1.0–4.3)*
Somnolence	1.05	2.76	5.67				16.8	6.8	5.44	*5.6 (3.4–6.5)*
Taste disturbance		1.06	1.34	4.1						*1.3 (1.2–2.7)*
Vomiting/nausea	8.9	4.38	2.11	7.7	4.9	2.55	12	10.5	8.18	*7.7 (4.4–8.9)*
Headache				9.9		10.59	27.4	19.9	18.01	*18.0 (10.6–19.9)*
Dizziness				5.1		1.81			5.5	*5.1 (3.5–5.3)*
Eye disorders				1.4			6.9	1.2		*1.4 (1.3–4.2)*
Constipation				6		6.85	10.7	4.2		*6.4 (5.6–7.8)*

^a^NSAID

^b^Triptans

^c^Anti-convulsant

**Table 3:** Adverse events (AE) in placebo groups compared with AEs in untreated groups

Study	Review in which study is contained	Patients reporting AEs (n)					
		No treatment		Placebo group		Treatment group	
		n	N	n	N	N	N
Barrett, 2010	Mahr, 2017	18	174	23	179	29	184
Bokmand, 2013	Koog, 2014a	1	34	8	29	5	31
Vas, 2012	Koog, 2014a	6	70	5	137	1	68
Choi, 2010	Koog, 2014a	1	14	2	15	2	15
Lee, 2009	Koog, 2014a	0	12	1	12	0	12
Sertel, 2009	Koog, 2014a	0	41	0	41	0	41
Friere, 2007	Koog, 2014a	0	12	0	12	0	12
Gioia, 2006	Koog, 2014a	0	25	0	25	0	25
Cabrini, 2006	Koog, 2014a	0	16	0	16	0	16
Melchart, 2005	Koog, 2014a	1	75	0	63	2	132
Linde, 2005	Koog, 2014a	2	76	1	81	4	145
Molsberger, 2002	Koog, 2014a	0	60	0	61	0	65
Leibing, 2002	Koog, 2014a	0	40	0	45	3	40
Medici, 2002	Koog, 2014a	0	20	0	23	2	23
Ma, 2010	Koog, 2014a	0	13	0	12	0	27
*Total*		*29*	*682*	*40*	*614*	*48*	*809*
*Percentage*		*4.25%*		*6.51%*		*5.93%*	

Fifteen trials within the reviews contained data about AEs reported in untreated groups (see Table 3). The average AE rate in these trials was smaller than the AE rate in placebo groups and treatment groups within the same trials (4.25%, 6.51%, and 5.93%, respectively). The AE rate in the placebo groups was higher than that of the untreated groups, although the difference was not statistically significant (*P* = 0.07), tentatively suggesting that the AE rates cannot be attributed to natural history. One study reported AEs within untreated groups, but not in a format we could pool [32]. In it, the authors found no significant differences between AEs reported in untreated, placebo, or treatment groups.

### Other sources of bias

Structured assessment seemed to result in different AE reports in some of the reviews (see “Additional analyses”).

### Additional analyses

Two reviews checked for differences between included trials with high versus low quality [12, 29]. Amanzio et al. [12] found that of 28 types of AEs measured, only one (somnolescence) showed a difference. Stathis et al. [29] found that the higher the trial quality (measured by Jadad scores), the lower the dropout rate in placebo groups.

Four reviews reported data about structured versus spontaneous reports of AEs [12, 14, 25, 26]. Only one of these found a statistically significant difference between AEs reported by different methods: Rief et al. [25] found it to be more than twice as likely for an AE to be reported using structured as opposed to spontaneous assessment methods (odds ratio 2.6, 95% confidence interval 2.1–3.3). For the 24 types of AEs where it was possible to compare spontaneous versus structured assessment, Amanzio et al. [12] did not find statistically different rates of AE reporting in 21 of them. Among the AEs where there was a difference, structured assessments resulted in higher prevalence of AEs for nausea in NSAID trials; spontaneous assessment resulted in greater AEs for fatigue in NSAID trials and nausea in Triptans trials. Hauser et al. [14] planned to compare AE rates for different assessment strategies but decided post hoc not to, due to poor quality of reports of assessment strategies; there did not appear to be a difference. Rojas-Mirquez et al. [26] reported which trials used different assessment methods but did not say whether there was a difference (and none were obvious by visual inspection).

## Discussion

### Summary

Our overview of systematic reviews of nocebo effects suggests that almost half of patients in the placebo groups within clinical trials experience AEs that are attributed to the drug. One in 20 patients in placebo groups drop out due to drug intolerance. This is higher than similar reports of AEs reported by patients taking the same interventions in routine clinical practice [14, 25].

### Comparison with other evidence

Our study adds to the existing reviews showing that AEs are common among patients in placebo groups within clinical trials. We showed that this phenomenon is common across several conditions and extends to many types of AEs. We also added a comparison of AEs in untreated groups, which suggested that natural history or misattribution is unlikely to explain the AEs reported within the placebo groups.

### Strengths and limitations

This is the first synthesis of AEs that covers both different disease areas and different types of placebos. It is also the first to investigate natural history as a potential cause of AEs within trial placebo groups. Our conclusions are limited by the quality of the included systematic reviews and the quality of the trials within the systematic reviews. This limitation is mitigated by our quality assessment of the included systematic reviews, which suggests that they meet a minimum quality standard. Another limitation is that a second extractor only reviewed a random selection of studies independently. This is unlikely to have had an important influence on the results as there were only five discrepancies, mostly about the nature of follow-up in untreated groups within individual trials.

It was also difficult to disentangle different possible causes of AEs within placebo groups. While our analysis did not reveal any clear trend in AE reports by ascertainment method (structured versus spontaneous), it remains possible that the way patients are asked to report AEs could influence what they report. This compounds the problem of distinguishing between effects of negative expectations and the effects of mistakenly attributing routine symptoms to a trial intervention. Hence, future experimental studies where expectations are manipulated are likely to be required to determine the potency of negative expectations. Relatedly, the included reviews did not define AEs, perhaps due to the failure of the included trials to do so.

### Implications for clinical trials and clinical practice

There are three implications of this study for trial methodology. First, methods to reduce the risk of AEs induced by negative expectations (that may arise due to the way possible harms are communicated) warrant investigation [33]. Some studies have already begun to address how clinicians might reduce nocebo effects within clinical trials, such as limiting suggestion of symptoms [34]. Identifying a model of informed consent that respects patient autonomy yet does not introduce unnecessary harm is therefore required. This might be achieved by shortening the informed consent process so that rare harms are made clear (for example, by being listed on a web page for patients who would like to know) but not forced upon patients. Oxman et al. proposed such a model [35], but it has not yet been implemented. Other ethicists have also called for “contextualizing” informed consent (adapting it for individual patients) [36]. Contextualized consent may enable practitioners to present benefits and harms to patients in ways that do not induce unnecessary nocebo effects. Second, we should modify the way AEs are collected such that natural history is not confused with AEs. This might be achievable with a combination of spontaneous and structured AE assessments.

Third, the inferences about intervention AEs needs to be rethought. This is typically calculated by comparing AE rates in treatment groups with AE rates in placebo groups. But if the AE rates in placebo groups are inflated by the way potential AEs are communicated to patients, this can lead to an overestimation of AEs in placebo groups and a consequent underestimation of AEs attributed to experimental treatments. The way AEs in trials are interpreted should therefore reflect the findings from this review.

Finally, our review also raises the ethical issue of balancing the need to respect patient autonomy and provide full informed consent, while at the same time reducing unnecessary harm done to patients. The former seems to demand that more information about AEs is provided, while the latter suggests that such information should be carefully communicated to avoid harms.

## Conclusions

Trial participants who receive placebos within clinical trials report experiencing AEs. Research on clinical trial methodology is now warranted to reduce this potentially unnecessary harm.

### Additional file

Additional file 1: **Table S1.** Review quality according to selected AMSTAR criteria. (DOCX 21 kb)

## Appendix

### PubMed search strategy

Search date: 22 November 2017

nocebo[TI] AND Review[ptyp]
