# Supplementary material for: Rapid overview of systematic reviews of nocebo effects reported by patients taking placebos in clinical trials
Source: Trials. 2018 Dec 11;19:674. doi: 10.1186/s13063-018-3042-4 (PMC6288933; doi:10.1186/s13063-018-3042-4)
Supplement: Supplementary file 1 — Table S1. Review quality according to selected AMSTAR criteria. (DOCX 21 kb) [file 13063_2018_3042_MOESM1_ESM.docx]

**Additional file 1**

**Additional methods**

Calculating adverse event rates within waiting list groups

One of the studies within the review (Barrett 2010) presented data about adverse events within waiting list groups, but not in a format that we could pool The authors of this study reported 5 types of individual adverse events (bad taste, diarrhea, headache, nausea, rash, stomach upset), and the number of participants that reported experiencing these events. They could not be combined since the same participant could experience more than one adverse event, which makes an accurate numerator difficult to compute. We therefore chose the adverse event that had the median percentage of adverse events in the untreated group (nausea).

**Table S1. Review quality according to selected AMSTAR criteria**

|  | **Quality assessment** | |
| --- | --- | --- |
| **Author** | **2 or more databases searched? (y/n)** | **Scientific quality of studies assessed and reported (y/n)** |
| Amanzio 2009 |  |  |
| *NSAID placebos* | y | y |
| *Triptans placebos* | y | y |
| *Anticonvulsant placebos* | y | y |
| Häuser 2012a |  |  |
| *fibromyalgia syndrome trials* | y | y |
| *painful diabetic peripheral neuropathy trials* | y | y |
| Häuser 2012b | y | n |
| Koog 2014a | y | y |
| Koog 2014b | y | y |
| Mahr 2017 | n | y |
| Mahr 2017 (additional) | ? | ? |
| Meister 2017 | y | y |
| Mitsikostas 2010 |  |  |
| *Symptomatic* | n | n |
| *Prophylactic* | n | n |
| Mitsikostas 2012 | n | n |
| Mitsikostas 2014 | n | y |
| Papadopoulos 2010 |  |  |
| *Symptomatic treatments* | n | n |
| *Disease modifying treatments* | n | n |
| Papadopoulos 2012 | n | n |
| Rief 2006 | n | n |
| Rief 2009 |  |  |
| *TCA studies* | y | y |
| *SSRI studies* | y | y |
| Rojas-Mirquez 2014 | y | y |
| Shafiq 2017 | n | n |
| Silva 2017 | y | y |
| Stathis 2013 | n | y |
| Zis 2015 | n | n |
| Zis 2017 | n | y |
